# Plant‐Based Anticancer Compounds With a Focus on Breast Cancer

**DOI:** 10.1002/cnr2.70012

**Published:** 2024-10-25

**Authors:** Amin Moradi Hasan‐Abad, Amir Atapour, Ali Sobhani‐Nasab, Hossein Motedayyen, Reza ArefNezhad

**Affiliations:** ^1^ Autoimmune Diseases Research Center Kashan University of Medical Sciences Kashan Iran; ^2^ Department of Medical Biotechnology, School of Advanced Medical Sciences and Technologies Shiraz University of Medical Sciences Shiraz Iran; ^3^ Department of Anatomy, School of Medicine Shiraz University of Medical Sciences Shiraz Iran

**Keywords:** adverse events, anticancer therapeutic, breast cancer, carcinogenesis, plant‐based compounds

## Abstract

Breast cancer is a common form of cancer among women characterized by the growth of malignant cells in the breast tissue. The most common treatments for this condition include chemotherapy, surgical intervention, radiation therapy, hormone therapy, and biological therapy. The primary issues associated with chemotherapy and radiation therapy are their adverse events and significant financial burden among patients in underdeveloped countries. This highlights the need to explore and develop superior therapeutic options that are less detrimental and more economically efficient. Plants provide an abundant supply of innovative compounds and present a promising new avenue for investigating cancer. Plants and their derivations are undergoing a revolution due to their reduced toxicity, expediency, cost‐effectiveness, safety, and simplicity in comparison to conventional treatment methods. Natural products are considered promising candidates for the development of anticancer drugs, due perhaps to the diverse pleiotropic effects on target events. The effects of plant‐derived products are limited to cancer cells while leaving healthy cells unaffected. Identification of compounds with strong anticancer properties and development of plant‐based medications for cancer treatment might be crucial steps in breast cancer therapy. Although bioactive compounds have potent anticancer properties, they also have drawbacks that need to be resolved before their application in clinical trials and improved for the approved drugs. This study aims to give comprehensive information on known anticancer compounds, including their sources and molecular mechanisms of actions, along with opportunities and challenges in plant‐based anticancer therapies.

## 1 Introduction

### 1.1 Cancer and Tumorigenesis

**FIGURE 1:**
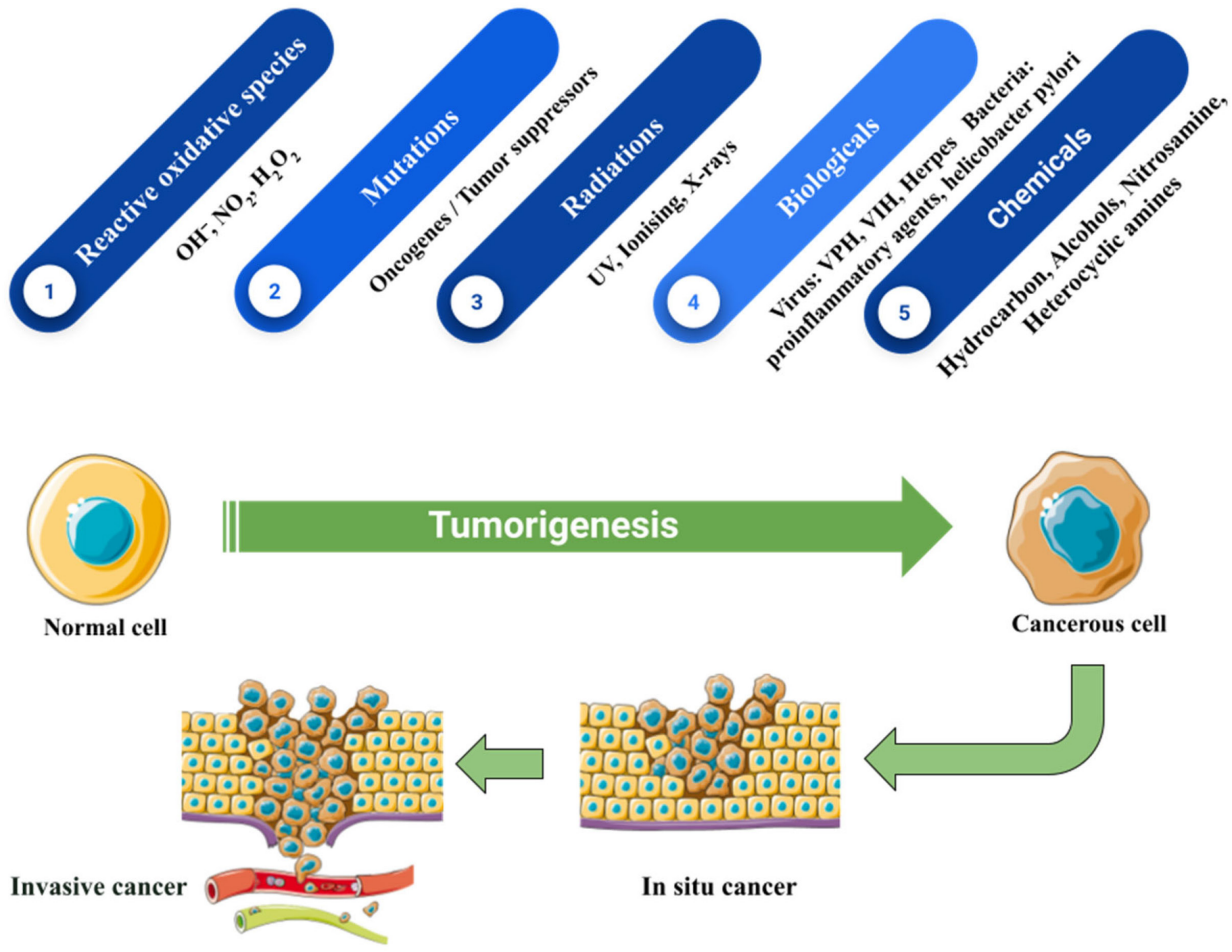
Main agents involved in the development of cancer.

A set of illnesses which are known as cancer involve abnormal cell proliferation and can infiltrate or spread to other regions of the body. In 2020, 18.1 million new cases of cancer occurred, which were 9.3 million male cases and 8.8 million female cases [1]. Its etiology is largely related to genetic, immunological, and environmental factors. Defects in immune responses, cell apoptosis, and DNA repair functions have fundamental roles in its development and progression [2, 3]. Oncogenes, as important genes involved in cell cycle regulation and apoptosis, can undergo genetic and/or epigenetically driven alterations that lead to cancer [4, 5]. The information provided by epidemiological studies is required to determine cancer risk factors (Figure 1). Random mistakes in DNA replication occurred at varying rates in various organisms are among the risk factors for developing cancer. Some environmental risk factors are responsible for developing cancer, including ionizing radiation, carcinogens, chronic infections, DNA damage response, metabolism, xenobiotic, immune system, hormone levels, and lifestyle elements such as smoking, hormone therapy, dietary consumption, and physical activity [6, 7].

The biological reactions to ionizing radiation are influenced by several factors including the type and energy of the radiation particle, the dose rate, the types of DNA damage, the cell type, and so on … . Ionizing radiation has the ability to damage nucleic acids, which could lead to alter the regulation of ordinarily expressed DNA [8, 9]. Some types of cancers have been linked to infections caused by specific organisms. For instance; stomach cancer is correlated to *Helicobacter pylori* infection. Gallbladder or colon cancer is linked with *Salmonella* infection. Liver cancer is related to *hepatitis C and B* infections. Kaposi sarcoma is correlated to the *herpes virus* infection. Cervical cancer is associated with the *human papillomavirus* infection [10, 11].

Toll‐like receptor pathways (TLR), nuclear factor‐kappa β (NF‐kβ), cGAS/STING, Janus kinase/signal transducers and activators of transcription (JAK–STAT), inflammation, and mitogen‐activated protein kinase (MAPK) have reported as the important factors related to the occurrence of cancer. Interferon (IFN), interleukins (ILs), tumor necrosis factor (TNF)‐like cytokines, chemokines, growth factors such as transforming growth factor (TGF), and vascular endothelial growth factor (VEGF) play key roles in cancer development. Some inflammatory metabolites, such as thromboxane, leukotrienes, prostaglandins, and specialized proresolving mediators (SPM), serve as significant regulators in the initiation and resolution of inflammation in cancer [12, 13].

### 1.2 Breast Cancer

Breast cancer, as the most prevalent malignancy and the primary cause of cancer‐related death among women worldwide, disrupts the lives of millions of women [14, 15]. In 2020, there were more than 2.3 million newly diagnosed cases of breast cancer and 685 000 fatalities resulting from the disease. There is significant variance in the incidence rates of this condition throughout different nations and areas of the world. In some Asian and African countries, the rates are below 40 per 100 000 women, whereas in Australia/New Zealand, Northern America, and parts of Europe, the rates exceed 80 per 100 000 women. Mortality rates showed less variance across different geographical areas. However, nations that are still transitioning continue to have a larger percentage of breast cancer fatalities compared to those that have already completed the shift. It is estimated that the incidence of breast cancer would annually reach more than 3 million new cases by 2040, resulting in 1 million fatalities per year alone due to population growth and aging [16]. For many years, breast cancer has consistently been ranked among the most lethal cancers in terms of incidence and mortality [17, 18, 19]. Similar to other cancers, it is influenced by a person's lifestyle, environmental circumstances, and genetic predisposition. Age and breast density are two examples of natural characteristics that can increase the risk of breast cancer. In addition, alterations in circadian rhythm, alcohol consumption, and tobacco chewing or smoking are associated with the increased risk of breast cancer [20]. These circumstances can result in cellular stress, the increased production of free radical oxygen species, and changes in progesterone and estrogen hormones, all of which enhance tumor aggression [21, 22]. Common therapies for breast cancer include chemotherapy, radiation therapy, and surgery; however, these methods have a poor prognosis and long‐term negative effects. Breast cancer therapy is impeded through metastasis, recurrence, and drug resistance, like treatment for other cancers [23, 24, 25]. Scientists are focusing on nutraceuticals as an emerging medicine with less side effects to address the issue with breast cancer treatment. Because of lower drug‐related adverse effects and resistance phenomena, nutraceuticals can also be used as an adjuvant therapy along with currently available chemotherapeutic medicines [26, 27]. Since ancient times, people have used natural remedies made from various plants or nonherbal sources to treat a variety of ailments, with many encouraging results. The needs for developing various herbal and nonherbal drugs with therapeutic potential are growing as a science and technology advance. As a result, there is plenty of room for innovative, healthy nutrition substrates. In 2017, it was estimated that the global market for nutraceuticals would reach 734 billion US dollars by 2026 [28, 29]. Therapeutic compounds, which are known as nutraceuticals have drug‐like features and are used to treat serious illnesses such as cancer, diabetes, atherosclerosis, neurological diseases, and hematological disorders. According to study, health food products contain polyphenols, terpenoids, tannins, alkaloids, and flavonoids, which have a significant potential to combat these fatal diseases [27, 30, 31]. In this review, specific important and pertinent breast cancer processes are highlighted, and nutraceuticals are assessed along with their processes and potential in breast cancer prevention.

### 1.3 Pathophysiology Involved in Breast Cancer

**FIGURE 2:**
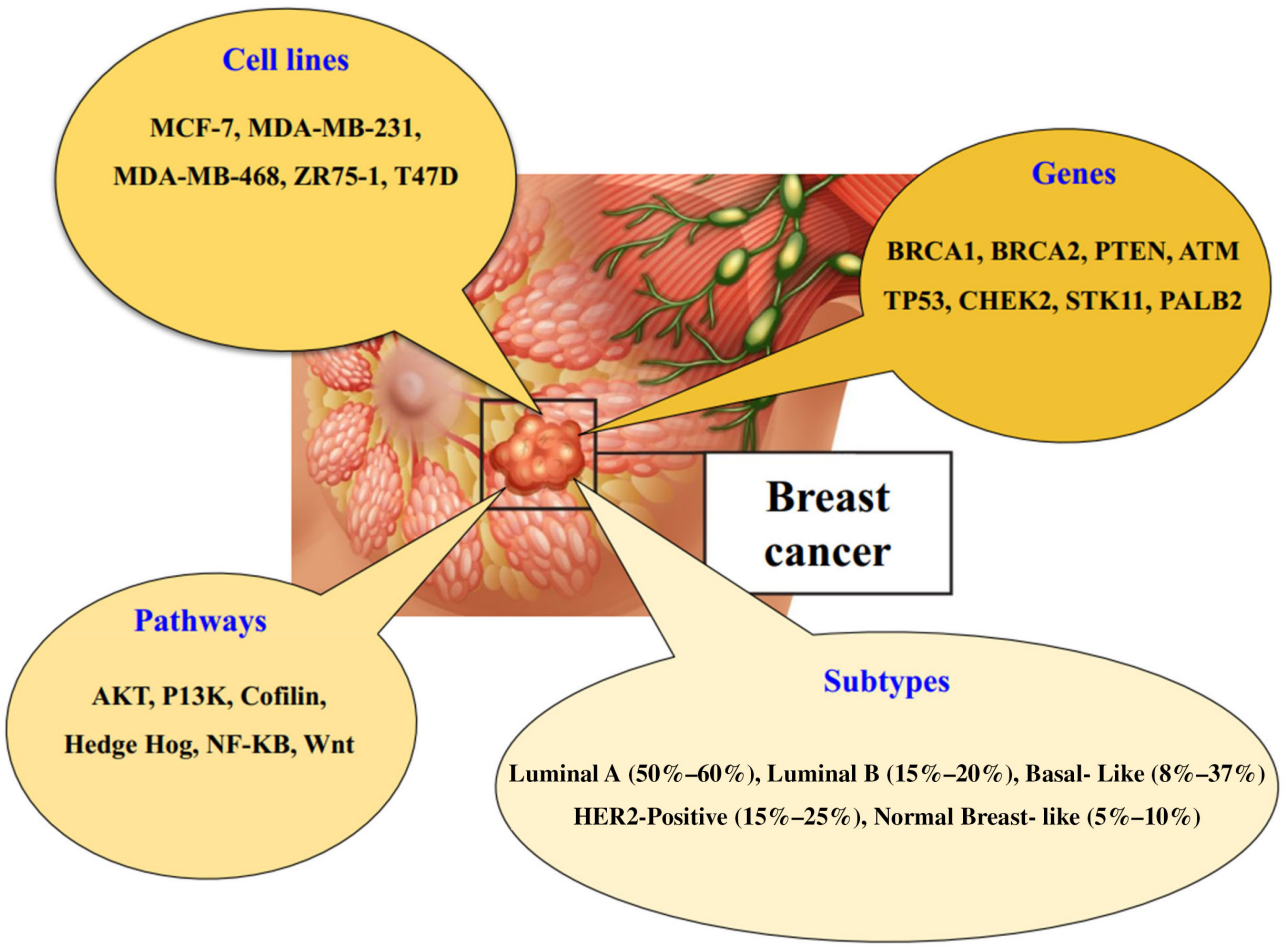
Classification, genetic factors, pathways, and cellular models of breast cancer.

Breast cancer typically starts as ductal hyperplasia and progresses through benign tumors and even metastatic cancer because of numerous toxins stimulating it. The effects of stroma and tumor microenvironment, including macrophages, play a critical role in the onset and progression of the cancer. Macrophages have the ability to create an immunosuppressive and mutagenic milieu that promotes angiogenesis and permits cancerous cells to spread [32]. The cancer stem cell and the stochastic theories are two hypotheses that may help to explain the development and spread of breast cancer. The stochastic hypothesis postulates that each tumor subtype is derived from a single cell type, differentiated progenitor, or stem cell. Any breast cell can get random mutations over time, and when enough mutations have accumulated, the cell eventually becomes a tumor cell. Based on the cancer stem cell theory, various tumor subtypes develop via identical progenitor cells or stem cells. Epigenetic and genetic alterations in precursor cells or stem cells have fundamental roles in determining different tumor features [33]. Numerous studies on tumor prognosis have identified programmed death‐ligand 1 (PD‐L1) as a biomarker for breast cancer [34]. Key risk factors for breast cancer include advanced age, a personal or family history of breast diseases and cancer, inherited genes such as *BRCA1* and *BRCA2* (which significantly increase the risk of breast cancer), as well as genes like *PTEN*, *ATM*, *TP53*, *CHEK2*, *STK11*, and *PALB2* (which carry a lower risk), exposure to radiation, and obesity [35, 36]. In order to create alternative treatments for breast cancer, it is crucial to increase a comprehensive understanding of the genes associated with different types of breast cancer and the specific cells from which they originate. This can be achieved through an immunohistochemical classification of receptor pathways involved in breast cancer, such as NF‐κβ, cofilin, Hedgehog, Akt, Wnt, PI3K‐Akt, PI3K‐Akt–mTOR, and PI3K [37, 38, 39]. The Akt pathway, which is suppressed in different types of malignancies, plays a crucial role in controlling cell proliferation and viability. An investigation of breast cancer tissue microarrays has revealed that the Akt pathway is active during the ductal carcinoma in situ (DCIS) stage, which is also known as stage 0 breast cancer [40]. The invasiveness and metastatic behavior of tumor cells are governed by the collective activity of the cofilin pathway. Cofilin, a widely distributed protein with a molecular weight of around 19 kDa, is present in invasive mammary carcinoma cells. Both breast cancer patients and those with mutations in the BRCA1 tumor‐suppressor gene exhibit a decrease in cofilin expression [41]. The aberrant activation of the Hedgehog (Hh) signaling pathway is specifically related to the formation and progression of cancer in several types of solid tumors. Overexpression of the Hh ligand is connected to the basal‐like subtype of breast cancer phenotype [42]. NF‐κβ as a transcription factor is expressed in all cells and plays a crucial role in the development of the mammary gland. It is linked to the advancement of estrogen receptor (ER)‐negative breast cancer. It is indicated that the p50 subunit of NF‐κβ, which binds to DNA, may serve as a prognostic marker to identify a subgroup of ER‐positive individuals at high risk [43]. The PI3K pathway has been shown to exhibit changes in several instances of breast cancer, resulting in the development of treatment resistance. Furthermore, it is shown that over 70% of breast tumors exhibit a mutation in at least one element of the PI3K pathway. This alteration might potentially be used to gain a therapeutic advantage in “basal‐like” malignancies [44]. Mutations in the *IK3CA* gene and activation of Akt via phosphorylation (pAkt) are often observed in many types of malignancies, with a particularly high occurrence in breast cancer. The preclinical and neoadjuvant trial results have indicated that a PIK3CA change exhibited resistance to HER2‐targeted treatment [45]. Figure 2 depicts different forms of breast cancer, specific genes that are involved, as well as the pathways and cell lines associated with the disease.

### 1.4 Limitations of Cancer Treatments and Anticancer Drugs

A growing body of studies has been performed to develop novel drug delivery and targeting methods, enhance drug accumulation and its efficacy, and minimize the negative side effects of medications during the course of cancer therapy [46]. The most current approaches to cancer treatment include surgery, radiotherapy, chemotherapy, immunotherapy, cancer vaccines, photodynamic therapy, stem cell transformation, and/or combinations of these options. These methods are largely related to serious side effects, including restricted metastasis, toxicity, nonspecificity, and reduced bioavailability [47, 48, 49]. Cancer treatments are dependent on the type, stage, and site of cancer. Cytotoxic and cytostatic drugs used in chemotherapy can exert their potential impacts alone and/or in combination with other forms of cancer therapy. Alkylating compounds, such as carboplatin, cisphamide, oxaliplatin, and melphalan, may lead to cardiovascular, gastrointestinal, hematologic, pulmonary toxicities, diarrhea, sensory neuropathy, and neutropenia [50, 51, 52]. These compounds are highly successful in treating numerous malignancies, but they have serious side effects, are expensive, complex, toxic, and unfriendly to the environment. Some cells, such as those located in the gastrointestinal tract (GIT), bone marrow, and hair follicles, develop quickly under usual physiological conditions. The contemporary anticancer medications also affect these rapidly growing healthy cells. These anticancer drugs can lead to GIT inflammation, hair loss, immunosuppression, cardiac conditions, reduction in blood production, and neurological issues (Table 1).

**TABLE 1:** Synthetic compounds with anticancer properties, their active compounds, and common side effects.

Drug classes	Active compounds	Mechanism of action	Common side effects^a^
Antimetabolites	5′‐Triphosphate, 5‐Fluorouracil, Deoxyadenosine, 1‐β‐D‐Arabinofuranoside, Mercaptopurine, Gemcitabine diphosphate and triphosphate, 5‐Fluorouracil, 9‐Beta‐D‐arabinosyl‐2‐fluoroadenine, 6‐Mercaptopurine, Methotrexate, 6‐Thioguanosine, 5‐Fluorouracil, Methyl‐tetrahydrofolate, Leucovorin	Antimetabolites disrupt the production of DNA components by mimicking the structure of purine and pyrimidine bases or folate cofactors, which play a role in purine and pyrimidine biosynthesis	Bleeding and bruising (thrombocytopenia), anemia, delirium loss, constipation, appetite, diarrhea, fatigue (swelling), edema
Alkylating agents	Busulfan, Carmustine, Acrolein and phosphoramide mustard, 5‐Aminoimidazole‐4‐carboxamide, Lomustine, Mechlorethamine, Melphalan, Azo‐Procarbazine, Triethylenethio‐phosphoramide, Semustine	Alkylating agents are chemicals that establish covalent connections with electron‐rich atoms in biological molecules. Traditionally, these agents are categorized into two types: those that directly react with biological molecules and those that create a reactive intermediate that then interacts with the biological molecules	Nausea and vomiting
Anthracyclines	Daunorubicin, Doxorubicin, Epirubicin, Idarubicin, Mitoxantrone	Anthracyclines induce DNA damage in cancer cells, leading to their death	Severe cough allergic, photosensitivity, hoarseness of voice, skin, and nail hyperpigmentation, flushing of the face, allergic reactions (anaphylaxis), fatigue, joint pain
Antitumor antibiotic	Bleomycin, Dactinomicyn, Mitomycin, Plicamycin	Antitumor antibiotic differ from antibiotics used for treating infections. They function by altering the DNA of cancer cells to inhibit their growth and reproduction. Anthracyclines are anti‐tumor antibiotics that disrupt enzymes responsible for DNA replication in the cell cycle	Feeling of unwellness (malaise) rash, fatigue, fever and chills, hair loss, diarrhea, nausea or vomiting, loss of appetite
Epipodophyllotoxins	Etoposide, Teniposide	Epipodophyllotoxins hinders DNA synthesis by creating a compound containing topoisomerase II and DNA. This complex causes breaks in double‐stranded DNA and inhibits repair by attaching to topoisomerase II. Accumulated breaks in DNA hinder progression into the mitotic phase of cell division, resulting in cell demise	Nausea, unusual tiredness or weakness, constipation, stomach pain, diarrhea, sores in the mouth and throat, loss of appetite or weight, vomiting
Taxanes	Cabazitaxel, Docetaxel, Paclitaxel	Taxanes inhibits cell growth by halting mitosis (cell division). Taxanes disrupt microtubule function	Fatigue at the IV site, muscle aches and pains, called myalgia, which can be extreme. redness or swelling, skin rashes, nausea and vomiting, hair loss, mouth sores, joint or bone pain
Vinca alkaloids	Vinblastine, Vincristine, Vinorelbine	Vinca alkaloids inhibit the division of cells in metaphase by attaching to tubulin and inhibiting its formation into microtubules	Nausea, diarrhea, constipation, tiredness/weakness, abdominal pain, headache, mouth sores, vomiting
Campotothecins	SN‐38 (7‐ethyl‐10‐hydroxycamptothecin), Topotecan	Camptothecin is thought to be a strong topoisomerase inhibitor that disrupts the crucial role of topoisomerase in DNA replication	Diarrhea, constipation, back pain, tiredness/weakness, headache, nausea, vomiting, abdominal pain
Platinum analogs	Carboplatin, Cisplatin, Oxaliplatin	They create interstrand and intrastrand cross‐links with DNA, which hinders DNA synthesis and transcription	Headache, vomiting, constipation, nausea, mouth sores, altered taste sensation, abdominal pain, diarrhea
Monoclonal antibody	Bevacizumab, Cetuximab, Rituximab, Trastuzumab	Monoclonal antibody target and identify certain proteins on cancer cells. They function in several ways to either eliminate the cancer cell or inhibit its growth	Nausea, allergic reactions such as hives or itching, diarrhea, including chills, fatigue, fever, muscle aches and pains, allergic reactions, vomiting, low blood pressure, skin rashes, flu‐like signs and symptoms
Growth inhibitor	Axitinib, Bortezomib, Sunitinib, Crizotinib, Lapatinib, Dasatinib, Imatinib, Dabrafenib, Nilotinib, Bosutinib, Sorafenib, Pazopanib, Trametinib, Vandetanib, Vemurafenib	Cancer growth blockers function by inhibiting the growth factors responsible for stimulating the division and proliferation of cancer cells	Coughing up blood, depressed mood, chest tightness. Clay colored stools, cloudy urine, bleeding gums, decreased urination, bloody nose

^a^ https://www.drugs.com/professionals.html.

It is reported that genetic conditions participate in the development of drug resistance in the cancer cells. *ABCA4* and *ABCA12* are mentioned as drug resistance genes related to breast cancer. Previous studies have indicated overexpression of these genes in human MCF‐7 cells after docetaxel treatment. However, their expressions were downregulated when the phytochemical curcumin was combined with docetaxel [53]. These observations suggest that cancer treatments need a combination of current therapeutic approaches. Consequently, plant‐derived compounds and related products may provide the most effective and safest methods for treating different cancers, based on the findings of many studies [54].

### 1.5 Plant‐Derived Anticancer Compounds

Newman and Cragg provide a full explanation of the functions of natural chemicals as medications or a foundation for the creation of new medications [5]. They found that 929 new drugs (antiviral, antifungal, antiparasitic, antibacterial, antitumor, etc.), approved in the last 40 years, had a natural origin. Approximately 29 of the 240 anticancer medications are purely synthetic, which may be due to natural compounds' benefits such as fewer side effects and the capacity to activate a variety of signaling mechanisms involved in cancer development. Additionally, during the preceding 10 years, synthetic compounds with natural pharmacological agents that mimic the actions of natural chemicals have been approved as anticancer medications [55].

In the realm of oncology, the use of herbal remedies has been extensively accepted as a supplemental or alternative treatment [56, 57]. Numerous new cytotoxic chemicals have been discovered from plants each year, opening up fresh avenues for the treatment of cancer. The study of naturally occurring molecular entities, which could be helpful to the pharmaceutical business is a focus for many academics [58]. When substances are found to have anticancer effects in preclinical research, researchers are often looking for a way to confirm their clinical efficacy. This review has a specific focus on breast cancer and describes the researches on herbal remedies with considerable and active anticancer activities as well as the anticancer ingredients discovered in such herbal treatments.

### 1.6 Breast Cancer Treatment With Plant‐Derived Anticancer Compounds

**FIGURE 3:**
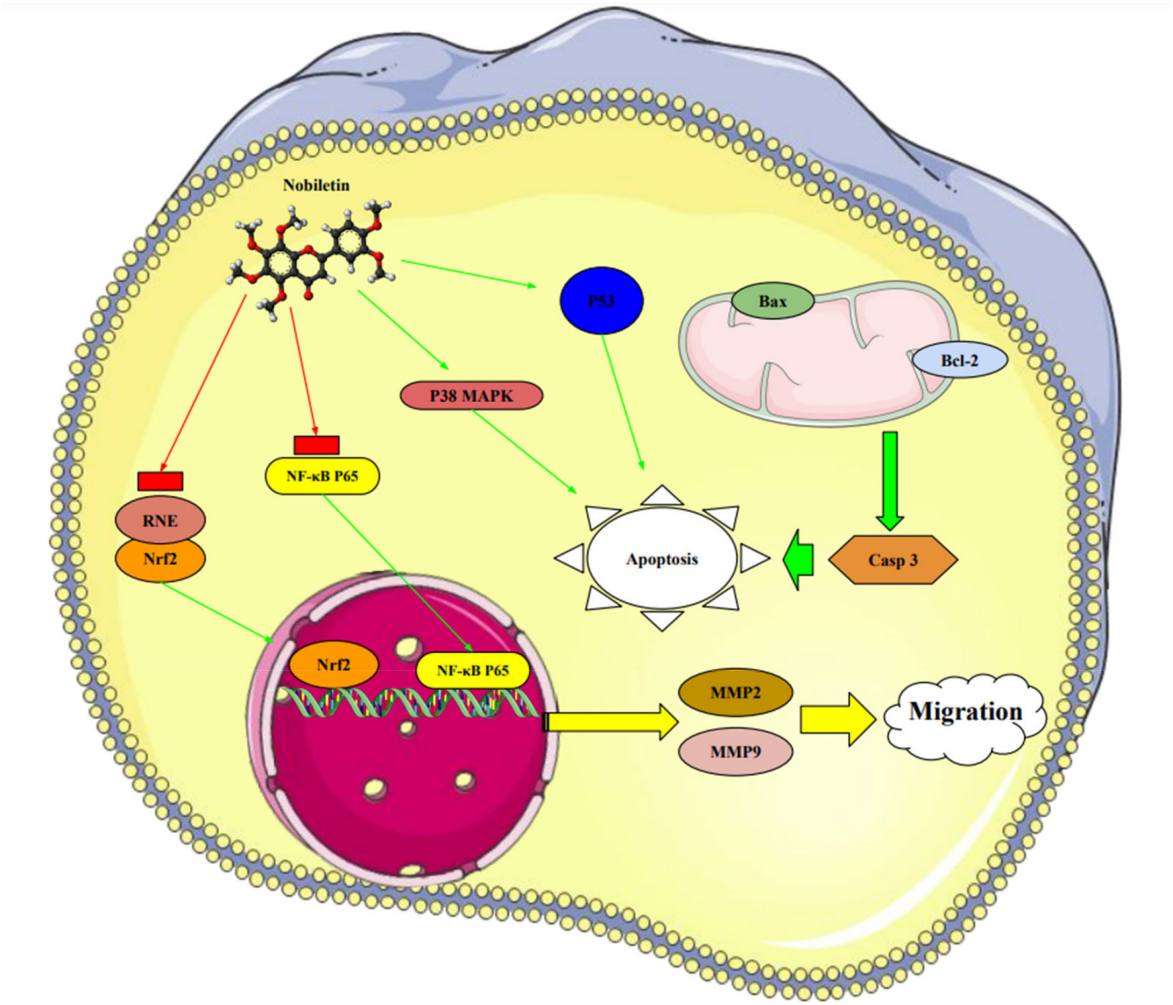
The plant derived‐natural compound (nobiletin) triggers programmed cell death and inhibits the movement and growth of human breast cancer cells through modulating the p38 MAPK, NF‐κB, and Nrf2 signaling pathways.

Nature provides various medicinal plants for humans to combat different diseases and improve public health. Since ancient times, people have used plants and their bioactive substances as medicines. Until now, numerous types of medicinal plants and their phytochemicals have been reported to avoid the spread and development of cancer [53]. There are over 250 000 plant species in the plant kingdom, but only about 10% of those have been studied for potential treatments of different diseases. Plant elements such as the flower, flower stigmas, pericarp, sprouts, fruits, seeds, roots, rhizomes, stem, leaf, embryo, and bark contain phytochemicals and their derived counterparts, which have a variety of therapeutic uses. Several primary and secondary metabolites play important roles in hindering cancer cell activating proteins, enzymes, and signaling systems or in activating DNA repair processes, promoting the formation of protective enzymes, and triggering antioxidant activity, resulting in potent anticancer effects. These metabolites include lignans, flavonoids, alkaloids, vitamins, terpenes, taxanes, saponins, mineral substances, oily substances, gums, glycosides, and biological molecules [59, 60]. In 2018, Liu et al. revealed that the molecular mechanisms of nobiletin are involved in reduction of the survival and proliferation of breast cancer MCF‐7 cells. Nobiletin causes apoptosis in MCF‐7 cells via altering the protein expression of Bax, Bcl‐2, cleaved caspase‐3, and p53. This compound exerts inhibitory impacts on Bcl‐2 expression and positive effects on Bax and p53 expressions in MCF‐7 cells. Furthermore, nobiletin reduced cell migration through decreasing the protein production of matrix metalloproteinase‐2 (MMP‐2) and matrix metalloproteinase‐9 (MMP‐9). Nobiletin's anticancer effects in MCF‐7 breast cancer cells may be mediated by the p38 mitogen‐activated protein kinase (p38 MAPK), NF‐κβ, and nuclear factor erythroid 2‐related factor 2 (Nrf2) pathways. It is revealed that nobiletin treatment participates in increased phosphorylation of p38 and decreased translocation of p65 and Nrf2 [61] (Figure 3).

**FIGURE 4:**
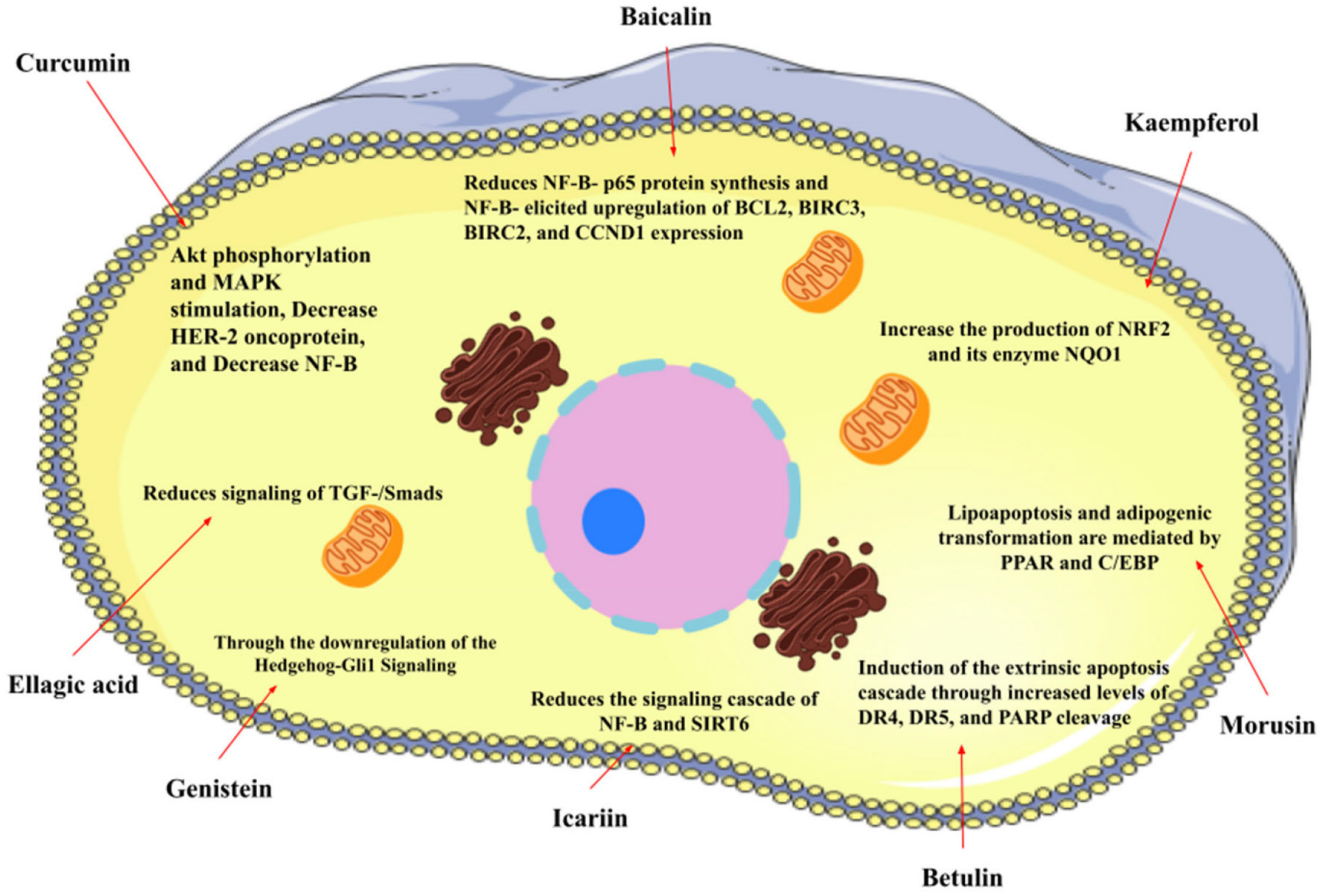
Anticancer mechanisms of some plant‐derived compounds in breast cancer model.

A study conducted by Espinosa‐Paredes et al., effects of *Echinacea angustifolia* (Ea‐AcOEt) extract on breast cancer cell lines were investigated. This compound has biological properties, including antiviral, immunomodulatory, and anticancer actions. The Ea‐AcOEt extract exhibited cytotoxic effects on breast cancer MDA‐MB‐231 cells (IC50 28 μg/mL) and MCF‐7 cells (20 μg/mL), while it did not have significant impacts on normal breast MCF‐10 cells. The Ea‐AcOEt extract mediates cell cycle arrest in G1 phase and triggers apoptosis via caspase activation. There is no evidence pointing to genotoxicity of the Ea‐AcOEt extract. In addition, animal studies failed to show toxicity and lethal effects of the Ea‐AcOEt extract at the dose of 2000 mg/kg in rats. In vitro studies have demonstrated that the combination of Ea‐AcOEt and paclitaxel extracts had a synergistic impact on breast cancer MDA‐MB‐231 cell and MCF‐7 cell lines. These findings suggest that *E. angustifolia* DC extract can be considered as a candidate for the treatment of breast cancer [30]. Actein, a compound derived from black cohosh (*Cimicifuga foetida*), blocks the Ras/MEK/ERK signaling pathway and inhibits Akt activation in MCF‐7 cells. It also prevents the phosphorylation of IKKα/β, and IKKα, which are proteins involved in the response to TNF‐α. Additionally, actein reduces the expression of genes that are downstream of NF‐κβ, a transcription factor associated with triple‐negative breast cancer (TNBC), which is a heterogeneous disease distinguished by unfavorable clinical outcomes resulting from inadequate response to standard therapies. KHF16, a cycloartane triterpenoid extracted from rhizomes of *C. foetida*, can result in G2/M phase arrest and apoptosis in MDA‐MB‐468 cells. It exhibited IC50 values of 5.6, 6.8, and 9.2 μM against MCF‐7, MDA‐MB‐231, and MDA‐MB‐468 cell lines, respectively [62, 63]. Hasasna et al. showed that *Rhus coriaria* had anticancer properties through inducing cell cycle arrest and autophagic cell death in metastatic triple negative MDA‐MB‐231 breast cancer cells. Additionally, the researchers assessed the impact of *R. coriaria* on the movement, infiltration, spread to other parts of the body, and proliferation of TNBC cells. It was indicated that *R. coriaria*, in concentrations nontoxic for the cells, effectively prevented migration and invasion, inhibited adherence to fibronectin, and reduced the levels of MMP‐9 and prostaglandin E2 (PgE2). *R. coriaria* not only reduced the attachment of MDA‐MB‐231 cells to human umbilical vein endothelial cells (HUVECs) and human pulmonary microvascular endothelial cells (HMVEC‐L), but it also hindered the movement of these cells via TNF‐α‐activated HUVECs. This study indicated that *R. coriaria* effectively suppressed the formation of new blood vessels (angiogenesis) and decreased the production of VEGF in both MDA‐MB‐231 cells and HUVECs. Additionally, it also reduced the expressions of pro‐inflammatory cytokines such as TNF‐α, IL‐6, and IL‐8. It is likely that *R. coriaria* exert its effects through blocking the NF‐κβ, STAT3, and nitric oxide (NO) pathways. Having considered that *R. coriaria* effectively regulates the development and spread of triple negative breast cancer, this compound may be considered as a very promising option for chemoprevention and therapy, [64]. Table 2 provides some information about some medicinal plants on cell lines of breast cancer. Moreover, the doses and therapeutic effects of natural compounds in animal models of breast cancer are summarized in Table 3. It is needless to say that numerous in vivo studies are required to assess in vitro impacts of natural compounds. In this regard, Table 4 indicates some clinical studies showing the effects of plant‐derived substances in breast cancer. Additionally, Figure 4 shows the generalized concept of carcinogenesis, immune responses, and efficiency of natural phytochemicals against cancer.

**TABLE 2:** Plant‐derived anticancer compounds in cell lines of breast cancer.

Natural compounds	PubChem CID and 2D structures	Chemical compositions	Cell lines	Active concentrations	Mechanisms of action	Ref
Nobiletin (*Citrus depressa*)	72344 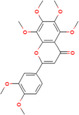	Bioactive polyphenol	MCF‐7	500 μL	Reduces ERK1/2, cyclin‐D1, p21 upregulation, mTOR, and AKT inhibition	[82]
Resveratrol (*Vitis vinifera*)	445154 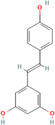	Phenol and a phytoalexin	4 T1	IC50 = 93 μM (72 h)	S‐phase slowdown cell cycle suppression, higher levels of apoptosis	[83]
			SUM159/MCF10A/MCF‐7
			100 mg/kg/d	Inhibition of Wnt β‐catenin pathway	[84]
/MDA‐MB‐231/MCF‐7
10, 25, and 50 μM	Decrease in MMP‐9, MMP‐2, c‐Myc, and cyclin D1 expression, decrease in Sox2 translation and stimulation of STAT3 and Akt	[85]
Curcumin (*Curcuma longa*)	969516 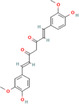	Phenolic	BT‐474/MDA‐MB‐231	1–25 μg/mL (72 h)	Akt phosphorylation and MAPK stimulation, decrease HER‐2 oncoprotein, and decrease NF‐κβ	[86]
			MDA‐MB‐231
			10, 20, and 30 μm/mL	Expression of EGFR and induction of cell death	[87]
			MCF‐7
1, 5, 10, 30, and 50 M	By suppressing NF‐B/AP‐1, MAPK, and PKC signaling, TPA causes MMP‐9 overexpression	[88]
MDA‐MB‐231/MCF‐10F/Tumor2
30 μM	Reduced expression of EMT‐associated, fibronectin, slug, N‐cadherin, Twist1, vimentin, AXL, and E‐cadherin/proteins‐catenin	[89]
Eugenol (*Syzygium aromaticum*)	3314 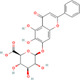	Allylbenzene	BT‐20/MDA‐MB‐231/MDA‐MB‐468	0.25, 0.50, 0.75, 1.0, and 1.5 μM	Inhibits NF‐B signaling, thereby decreasing IL‐8 and IL‐6 production	[90]
Baicalin (*Scutellaria baicalensis*)	64982 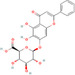	Phenolic	MCF‐7/MDA‐MB‐231	0.20 or 30 μM	Reduces NF‐B‐p65 protein synthesis and NF‐B‐ elicited upregulation of BCL2, BIRC3, BIRC2, and CCND1 expression	[91]
Artemisinin (*Artemisia annua*)	68827 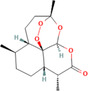	Alkaloid	MDA‐MB‐231	25, 50, and 100 μM (48 h)	Reduce Bcl‐2, enhance Bax, G2/M‐phase arrest reduce cyclin‐B1 and cyclin‐D1, heterochromatin agglutination, degeneration of mitochondrial vacuoles; nuclear enlargement, reduce the amount of organelles within cells	[92]
Ellagic acid (*Juglans regia*)	5281855 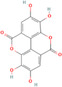	Polyphenolic	MCF‐7 cells	0, 10, 20, and 30 μg/mL	Reduces signaling of TGF‐/Smads	[93]
Ginseng extract (*Ginseng*)	—		MCF‐7	100–400 μM (24 h)	Reduce Bcl‐2, augment Bax, cytochrome c, and activated caspase‐3; augment ROS production	[94]
Oleuropein (*Olea europaea*)	5281544 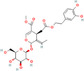	Phenolic bitter	MCF‐7	1.7 mg/day	Blocking the growth and proliferation of MCF‐7 cell xenografts	[95]
Eupatorin (*Eupatorium semiserratum*)	97214 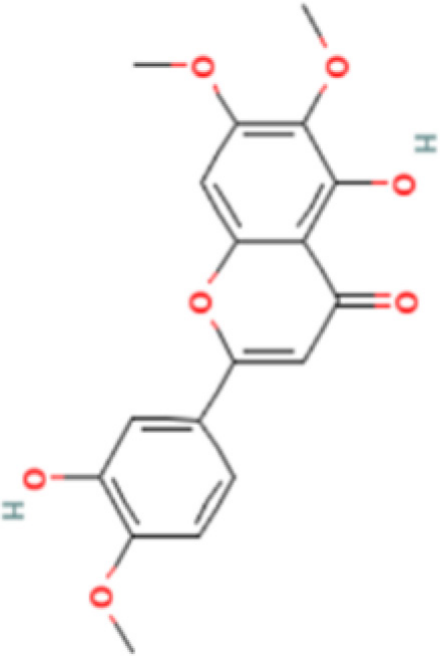	Flavone	MCF‐7/MDA‐MB‐231	20 μg/mL	By inhibiting the p‐Akt pathway, and boosting the production of SMAC/Diablo, cytochrome c, Bax, Bak1, Bad, and HIF1A	[96]
Emodin (*Aloe vera*)	3220 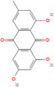	Anthraquinone	—	IC50 = 8.6 μM	Unique energy‐dependent pathway of drug uptake inducing apoptosis	[97]
Genistein (*Glycine max*)	5280961 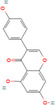	Isoflavone	MCF‐7	10 nM–10 mΜ	Through the downregulation of the Hedgehog‐Gli1 signaling	[98]
			MCF‐7/MDA‐MB‐231/MCF10a
18.5 μM	Elevated levels of BRCA2 and BRCA1 protein	[99]
Artesunate (*Artemisia annua*)	6917864 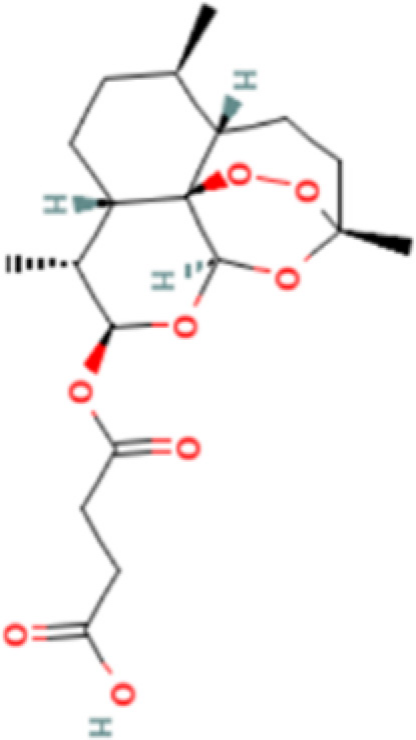	Hemisuccinate ester of the lactol	—	IC50 = 2.3 μM	VEGF expression reduction	[100]
Kaempferol (*Moringa oleifera*)	5280863 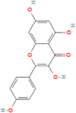	Flavonoid	MDA‐MB231	50 μM	Increase the production of NRF2 and its enzyme NQO1 in MCF‐7 cells, thereby preventing oncogenesis	[101]
			MCF‐7
10 μM	Halts cell cycle progression at the G2/M phase by inhibiting CDK1	[102]
Betulinic acid (*Betula* sp.)	64971 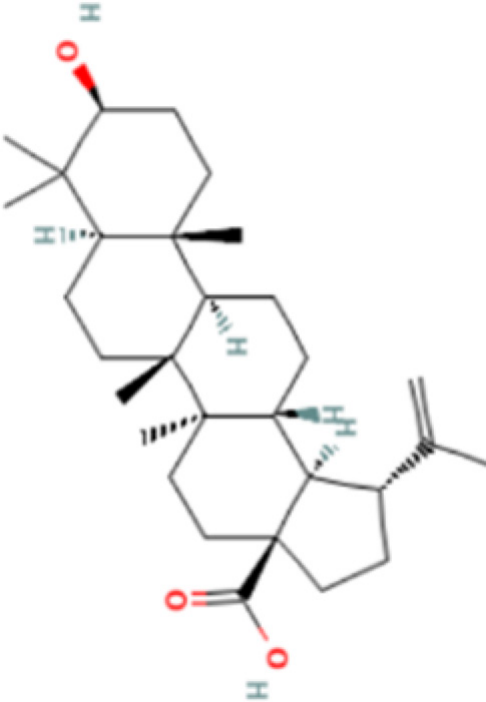	Pentacyclic triterpenoid	—	IC50 = 13.5 μM	Induction of the extrinsic apoptosis mechanism through increased levels of DR4, DR5, and PARP cleavage	[103]
Icariin (*Epimedium sagitatum*)	5318997 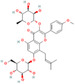	Flavonoid	MDA‐MB‐231/4 T1	10 or 20 μM	Reduces the signaling cascade of NF‐B and SIRT6	[104]
Betulin (*Betula* sp.)	72326 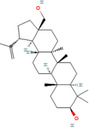	Pentacyclic lupane‐type triterpenes	—	IC50 = 30.7 μM	Induction of the extrinsic apoptosis cascade through increased levels of DR4, DR5, and PARP cleavage	[105]
Isoliquiritigenin (*Spatholobus suberectus*)	638278 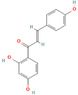	Phenolic	MCF‐7/MDA‐MB‐231/BT‐549	10 μg/mL	By blocking β‐catenin/ABCG2 signaling	[106]
Berberine (*Berberis vulgaris*)	2353 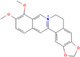	Benzylisoquinoline alkaloids	—	IC50 = 25 μM	Activation of cell cycle arrest, a combined effect with drugs/dose‐dependent decrease in tumor volume and angiogenesis	[107]
Epicatechin gallate (*Camellia sinensis*)	107905 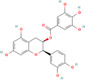	Polyphenol	—	IC50 = 350 μM	Promote apoptosis in various types of cells of cancer	[108]
Morusin (*Ramulus mori*)	5281671 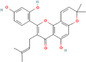	Flavonoid		1, 2, 4, 6, and 8 μg/mL	Lipoapoptosis and adipogenic transformation are mediated by PPAR and C/EBP	[109]
Epigallocatechin (*Camellia sinensis*)	65064 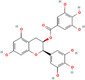	Polyphenol	—	IC50 = 22 μM	Growth restriction	[110]
D Rhamnose (*Clematis ganpiniana*)	5460029 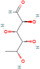	Triterpenoid saponins, favonoids, coumarins, alkaloids	MCF‐7/SUM1315/	5, 10, 20, 40, and 80 μg/mL	Via inhibiting the pathway of PI3K and AKT and enhancing the ERK pathway	[111]
β‐Hederin (*Clematis ganpiniana*)	441929 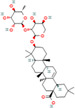	Triterpenoid saponin	MDA‐MB‐231/BT474	5, 10, 20, 40, and 80 μg/mL	Via inhibiting the pathway of PI3K and AKT and enhancing the ERK pathway	[111]
Myricetin (*Camellia sinensis*)	5281672 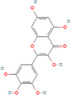	Flavone	MDA‐Mb‐231	50 mg/kg	By suppressing the expression of MMP2/9 and ST6GALNAC5 proteins	[112]
Ingenol mebutate (*Euphorbia peplus*)	137795436 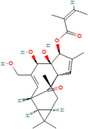	Ester	—	IC50 = 23.9 μM	Necrotic mechanism	[113]
β‐Elemene (*Rhizoma zedoariae*)	6918391 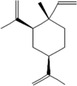	Sesquiterpene	MDA‐MB‐231/MCF‐7	0–320 μM/L	Inhibiting the aerobic glycolysis triggered by dimer formation of PKM2 and nuclear transfer	[114]
Dehydrocorydaline (*Corydali syanhusuo*)	34781 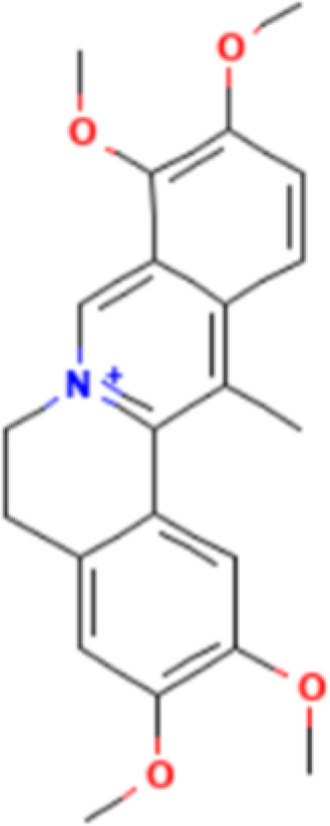	Alkaloid	MDA‐MB‐231	20,30, 40, 50, or 100 μM	By reducing BCL 2, CCND 1, BCL 3, and CDK1 and enhancing the generation of pro‐apoptotic proteins caspase9/3/8	[115]
Theacrine (*Theobroma grandiflorum*)	75324 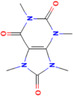	Purine alkaloid	MDA‐MB‐231	10–100 μM	EMT induced by TGF is inhibited	[116]
Bilobetin (*Ginkgo biloba*)	5315459 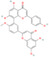	Flavonoid		IC50 = 57.6 μM	Stopping the cell cycle at the G2/M phase	[117]
Harmine (*Peganum harmala*)	5280953 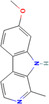	Alkaloid	MDA‐MB‐231 and MCF‐7	50, 100 or 150 μM	By decreasing pErk, Bcl2, pAkt, and TAZ expression	[118]
Isoginkgetin (*Ginkgo biloba*)	5318569 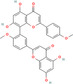	Biflavonoid	—	IC50 = 92.1 μM	Stopping the cell cycle at the G2/M phase	[117]
α‐Santalol (*Santalum album*)	482129503 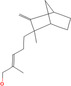	Sesquiterpene	20, 40 μM	MDA‐MB 231/MCF‐7	Via suppressing the Wnt/−catenin signaling pathway	[119]
Licoagrochalcone (*Glycyrrhiza glabra*)	11 099 375 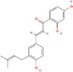	Phenol chalconoid	—	IC50 = 28.6 μM	Promotion of apoptosis and suppression of cell division	[120]
Astragaloside IV (*Astragalus membranaceus*)	13943297 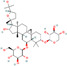	Pentacyclic triterpenoid	MCF‐7/MDA‐MB‐231//MDA‐MB‐468	40 and 80 μg/mL	Prevent BC cell proliferation and metastasis via inducing expression of TRHDE‐AS1	[121]
Apigenin (*Matricaria chamomilla*)	5280443 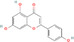	Flavone	—	IC50 = 100 μM	Augmentation of the DR5 mechanism	[122]
Betulinic acid (*Castanopsis acuminatissima*)	64971 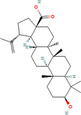	Triterpenoid	MCF‐7	50 μM	Via reducing topoisomerase or cyclin, suppressing VEGF signaling, decreasing SP and NF‐B stimulation, and downregulating matrix metalloprotease production	[123]
Chamomillol (*Matricaria chamomilla*)	91747197 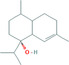	Terpene	—	IC50 = 300 μM	Repress angiogenesis by repressing expression proteins	[124]
Cucurbitacin B (*Cucurbeta pepo*)	5281316 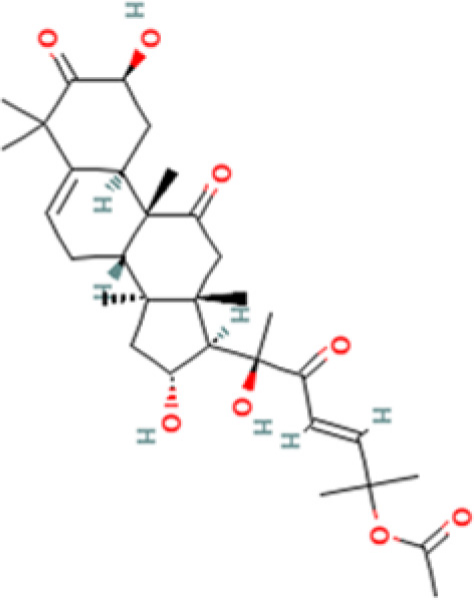	Cucurbitacin	MDA‐MB‐231	0.5 and 1 mg/kg	Reduce NF‐B and STAT3	[125]
Lycorine (*Narcissus pseudonarcissus*)	72378 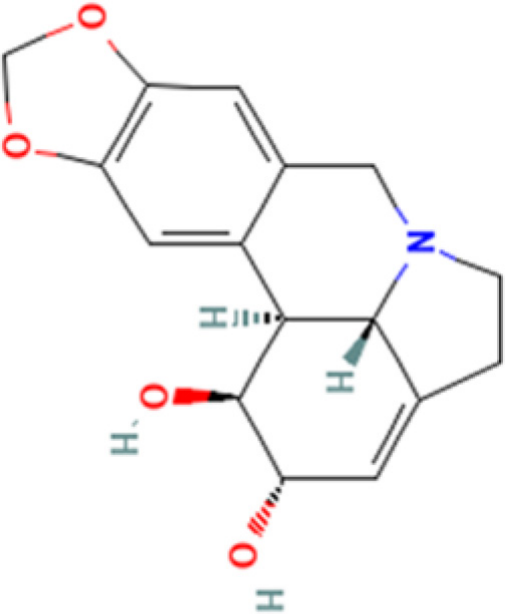	Alkaloid	MCF‐7/T47D/MDA‐MB‐231	5 or 10 mg/kg	Via interfering with the Src/FAK pathway	[126]
Ginsenoside (*Panax ginseng*)	3086007 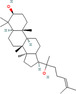	Triterpene saponin	—	IC50 = 30 μM	Activation of apoptosis and suppression of cell division	[127]
Citral (*Cymbopogon citratus*)	638011 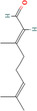	Acyclic monoterpene aldehyde	MDA‐MB‐231	2.5, 5.0, and 10.0 μg/mL	Reducing the expression of aldehyde dehydrogenase 1A3	[128]
Borbonol (*Persea americana*)	10448019 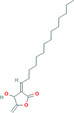	Isoalkane	—	IC50 = 20.5 μM	Inhibition of proliferation	[129]
Matrine (*Sophora flavescens*)	91466 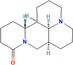	Tetracyclic quinolizidine alkaloid	MCF‐7	2, 4, 8, and 10 mM	Through the aggregation of light chain 3 II and reduced levels of p62, phosphorylation of mTOR and AKT was suppressed	[130]
Germacrone (*Rhizoma curcumae*)	6 436348 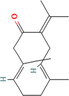	Sesquiterpene	MCF‐7	100 or 200 μM	Via inhibiting ER‐driven gene expression	[131]
Salvicine (*Salvia prionitis*)	10359290 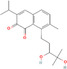	Quinone		IC50 = 1.4 μM	Breaks two strands of DNA through increasing TOP2 activity; prevents re‐ligation	[132]
Noscapine (*Papaver somniferum*)	275196 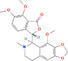	Benzylisoquinoline alkaloid	MCF‐10/MCF‐7/MDA‐MB‐231	20, 40, 60, 80, and 100 μM	A decrease in the expression of the NF‐B gene and protein, along with an increase in the expression of the IB gene	[133]
Cepharanthine (*Stephania cepharantha*)	10206 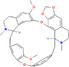	Isoquinoline	MDA‐MB‐231 and MCF‐7	5 μM for MCF‐7, 4 μM for MDA‐MB‐231	By disrupting AKT/mTOR signaling system	[134]
Ursolic acid (*Ocimum tenuiflorum*)	64945 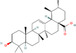	Pentacyclic triterpenoid	MDA‐MB‐231	10.0 μg/mL	Reduced EGFR, PI3K/Akt/mTOR, and ERK activity	[135]
Protocatechu aldehyde (*Salvia miltiorrhiza*)	439260 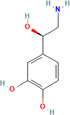	Protocatechuic aldehyde	MCF‐7 and MDA‐MB‐231	0, 5,10, 25, 50, or 100 lM	Inhibits the expression of—catenin and cyclin D1	[136]
Silibinin (*Silybum marianum*)	31553 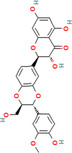	Aldehyde	—	IC50 = 24 μM	Apoptosis induction and cell cycle arrest	[137]
Piperlongumine (*Piper longum*)	637858 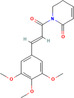	Aldehyde	MCF‐7/MDA‐MB‐231/MDA‐MB‐453 and BT‐549	6.25,12.5, 25, 50, and 100 lM	Through decreasing Bcl‐2, cyclin D1, p‐Akt, p53, p70S6K1, and 4E‐BP1 expression and increasing cytochrome c and Bax expression; by inhibiting the PI3K/Akt/mTOR signaling axis	[138]
Gossypol (*Gossypium hirsutum*)	3503 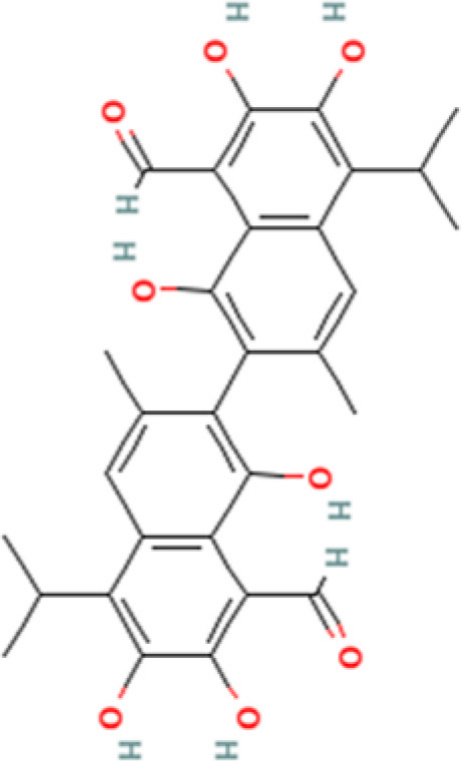	Aldehyde, ketone, lactol	MDA‐MB‐231/MDA‐MB‐468	0–100 μM	Increases the expression of BNIP3, TNFRSF9, and GADD45A	[139]
			MM‐231/MM‐468			
0–50 μM
Silymarin (*Silybum marianum*)	5213 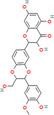	Aldehyde	—	IC50 = 75 μM	Interaction with the production of cell cycle regulators and apoptotic proteins; activation of cell cycle arrest	[140]
Pterostilbene (*Cyanococcus*)	5281727 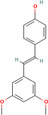	Phenol	MDA‐MB‐468	Dose: 50 μM	Reduces phosphorylation of mTOR and AKT and decreases cyclin D1 expression	[141]
Paradol (*Zingiber officinale*)	94378 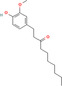	Phenol	—	IC50 = 20.4 μM	Reduced cellular viability	[142]
Colchicine (*Colchicum autumnale*)	6167 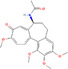	Alkaloid	MCF‐7	0.3125, 0.625, 1.25, 2.5, 5, 10, 20, and 40 μg/mL	Through cells unable to exit the G2/M phase	[143]
Tanshinone‐IIA (*Salvia miltiorrhiza*)	164676 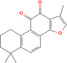	Aldehyde	MDA‐MB‐231	0, 3, and 6 μM	Through inducing S‐phase cell cycle arrest and boosting GSK3 expression, MMP expression is inhibited	[144]
Shogaol (*Zingiber officinale*)	5281794 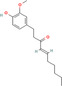	Aldehyde	—	IC50 = 24.4 μM	Inhibitory activity	[145]
β‐caryophyllene oxide (*Myrica rubra*)	482135 081 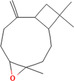	Bicyclic sesquiterpene	MDA‐MB‐231 and MCF7	5–500 μM	Inhibition of NF‐κβ	[146]

**TABLE 3:** Plant‐derived anticancer compounds in animal model of breast cancer.

Natural compounds	Source extractions	Animal models	Dose/administrations	Therapeutic effects	Ref
EGCG	Epigallocatechin gallate	CB‐17 rodents with severe combined immunodeficiency	100 mg/kg of EGCG dissolved into 100 L of water is administered orally every 2 days	Reduce tumor proliferation; reduce miR‐25 expression; reduce Ki‐67; and enhance pro‐apoptotic PARP expression	[147]
Artemisinin (Artesunate)	*Artemisia annua*	Xenograft model of 4 T1 cells in female BALB/c mice	Every day intraperitoneal injection of 100 mg/kg artemisinin dissolved in 0.2% DMSO for 20 days	Reduced splenic and tumor Treg and MDSC growth; enhanced percentages of CD4+ IFN‐ +T cells; elevated FN‐ and TNF—	
Ginseng	Ginseng	Xenograft model of MCF‐7 cells in female BALB/c athymic nude mice	Ginseng extract (50 or 100 mg/kg) was given intravenously once a day for 4 weeks	Increase Bax, activated caspase‐3, and activated PARP; reduced Bcl‐2; reduced tumor weight	[94]
Resveratrol	Skin of grapes, blueberries, raspberries, mulberries, and peanuts	Xenograft model of MDA‐MB‐231 cells in female athymic mice	Resveratrol (ethanolic solution) 25 mg/kg/day intraperitoneally for 3 weeks	Reduce tumor size, boost apoptotic index, and stop angiogenesis	[148]
Curcumin	Turmeric or *Curcuma longa*	Female athymic nude mice with BT‐474 xenograft mod‐el overexpressing HER‐2	Curcumin was administered intraperitoneally twice weekly for a period of 4 weeks at a dose of 45 mg/kg, dissolved in 0.1% DMSO	Tumor volume reduction	[86]
Berberine	*Berberis vulgaris*	—	IC50 = 25 μM	Cell cycle arrest induction, drug–drug interactions, dose‐dependent tumor volume decrease, and angiogenesis	[107]
Combretastatin	*Combretum caffrum*	Mice	IC50 = 80–190 μM	Tubulin binding results in the microtubules being less stable	[149]
Ginkgetin	*Ginkgo biloba*	Mouse	IC50 = 10 μM	Autolysosome production and redox environment are mediated by p62/SQSTM1, and the signaling transducer and activator of transcription 3 activity is inhibited	[150]
Noscapine	*Papaver somniferum*	Mice	IC50 = 45 μM	Triggering several signaling cascades, such as apoptosis	[151]
Cryptotanshinone	*Salvia prionitis*	Mice	IC50 = 1.1 μM	Multifaceted mechanisms of action include apoptosis, G2/M arrest, and cellular movement inhibition. All these pathways are orchestrated by NFB inhibition	[152]

**TABLE 4:** Clinical studies on plant‐derived anticancer substances for breast cancer.

Natural compounds	Identifiers	Titles	Observations
Resveratrol	NCT04266353	Effect of resveratrol on serum IGF2 in African American women	Participants will be given 150 mg of resveratrol per day for 6 weeks
Ginseng	NCT00631852	A phase II biomarker trial of gelatin encapsulated extract of American ginseng root (LEAG) in breast cancer	American ginseng extract from the roots was administered as follows: four 250 mg tablets were taken daily for 5–14 days prior to surgery
Artemisimin (Artesunate)	NCT00764036	Patients with metastatic or locally advanced breast cancer are being studied in a prospective open uncontrolled phase I study to determine the compatibility, safety, and pharmacokinetics of the semi‐synthetic artemisinin derivative artesunate from the Chinese herb *Artemisia annua*	The medication was given orally once day for 4 weeks in dosages of 100, 150, or 200 mg of artesunate
EGCG	NCT00917735	Study of the green tea extract's efficacy on breast cancer risk biomarkers in high‐risk women with different catechol‐o‐methyl transferase (COMT) genotypes under placebo control	Two green tea extract capsules containing 51.7% EGCG should be taken orally twice day, after breakfast and supper, for a period of 1 year
Curcumin	NCT03980509	Curcumin, the active ingredient in turmeric, in a “Window Trial” for primary invasive breast cancer tumors	From the time surgical resection is scheduled until the night before surgical resection, 500 mg of curcumin will be given orally twice daily, after each meal

### 1.7 Breast Cancer Treatment With Plant‐Derived Extracellular Vesicles

Small extracellular particles are membrane‐bound vesicles (EVs) released by the cells and have the ability to contain a variety of biological macromolecules, such as metabolites, proteins, and nucleic acids. EVs are a new breed of natural drug delivery methods because of their low immunogenicity, high biocompatibility, and the ability to load functional modules into their payload. However, the limited yield and high manufacturing cost of EVs produced from animal cells reduce their practical utility [65]. On the other hand, plant‐derived EVs, or EPDEVs, are gaining more and more interest since they can be made in vast numbers from plant sap. Recent studies have revealed the therapeutic potential of plant‐derived extracellular hoover cleaners in tissue healing, anti‐inflammatory, and antitumor effects, which raises the possibility of EPDEVs as a ground‐breaking nanotherapeutic system [66, 67].

In order to prevent tumor cells from growing, Raimondo et al. showed that EVs in citrus lemon juice could induce the expression of TNF‐related apoptosis‐inducing ligand (TRAIL), increase the expression of pro‐apoptotic genes (*Bad* and *Bax*), and decrease the expression of anti‐apoptotic genes (*Survivin* and *Bcl‐xl*). These effects exerted without damaging normal cells [68]. In line with prevention of angiogenesis, it was able to downregulate the production of VEGF‐A, IL‐6, and IL‐8. These observations suggest that lemon ELNs may prevent the growth of cancer cells through enhancing TRAIL‐mediated apoptosis and preventing the release of VEGF‐A, IL‐6, and IL‐8. It is reported that ginseng‐derived vesicles are rapidly recognized and taken up by macrophages, causing M1 polarization and encouraging reactive oxygen species (ROS) production, which thereby participating in the apoptosis of mouse melanoma cells and suppressing the formation of tumors [69]. Ginger‐derived EVs, also known as grapefruit‐derived lipid nanoparticles or GDNV, may be employed as nanocarriers to deliver 5‐fluorouracil (5‐FU) for the treatment of colon cancer [70]. The combination of modified GDNVs with the targeting ligand folic acid can facilitate the delivery of 5‐FU to the colon‐26 tumors in vivo.

In order to address the poor stability and limited drug binding capacities of EVs, Saroj et al. produced polymeric drug‐extracted vectors (PDEVs) from curry, neem, and mint leaves coupled with chitosan (CS) and PEGylated graphene oxide (GP), which transformed them into strong and effective vectors. The engineered conjugates effectively injected MCF7 breast cancer cells with siRNA that targets the estrogen receptor α (ERα1). Previous studies have indicated that neem‐based EV‐CS‐GP conjugates are the most effective in delivering siRNA inside cells. It is likely that MCF7 cells recognize neem EVs through CD44 receptors and hyaluronic acid [71]. This method demonstrates a cutting‐edge strategy to creating environmentally friendly, nontoxic, and therapeutically feasible EV‐based cars that can transport a range of useful siRNA cargos [71].

Using 2D and 3D cell culture, Cui et al. examined the anticancer effects of CLENs on TNBC cell line (4T1 and HCC‐1806 cells). They discovered that CLENs were taken up by the cells via endocytosis, which resulted in a time‐ and dose‐dependent reduction in cell survival [72]. Furthermore, it is reported that 40 and 80 μg/mL concentrations of CLENs significantly suppressed the migration and evasion of TNBC cell line. These findings propose that CLENs are effective against TNBC cell line [72]. Moreover, some distinct benefits of CLENs, including its high stability, inherent bioactivity, and high absorption, make them as a natural drug delivery method. Therefore, underpin PDEVs' functionality may open up new avenues for the treatment of human diseases like breast cancer [73]. However, further studies are required to clarify their molecular processes and biological elements.

### 1.8 Combination Therapy With Plant‐Derived Substances in Breast Cancer

Chemotherapy is still an unavoidable vital tool for breast cancer treatment, even with significant advancements in cancer treatment. Consequently, reduction in chemoresistance and chemotherapy side effects is crucial. Many attempts have been undertaken in this field.

It is revealed that overexpression of Flap endonuclease 1 (FEN1) has an important role in development of breast cancer. In this regard, previous reports have revealed that Cisplatin triggers ERK/MAPK pathway in breast cancer cells. In turn, Cisplatin‐activated ERK stimulates transcription factors that attach to the FEN1 promoter. This ultimately increases the synthesis of FEN1 protein, and FEN1 overexpression enhances the capacity of DNA repair, leading to promote resistance to Cisplatin treatment. In another study conducted by Zou et al., synergistic effects of the Curcumin and Cisplatin on breast cancer cells were investigated. It was shown that Curcumin plus Cisplatin increased the susceptibility of breast cancer cells to cisplatin through downregulating *FEN1* expression both in vivo and in vitro. In fact, Curcumin downregulated the expression of *FEN1* through reducing the phosphorylation of ERK and inactivation of specified transcription factors, which are able to bind to the FEN1 promoter. Subsequently, Curcumin decreases the capacity of DNA repair and enhances the susceptibility of breast cancer cells to cisplatin [74]. Regarding synergistic effects of plant‐derived substances, nobiletin in combination with docetaxel (DTX) or carboplatin (CAR) inhibits the proliferation of TNBC cells. Furthermore, the efficacy of nobiletin in combination with DTX or CAR on the proliferation of MDA‐MB‐231 cells was assessed. Kim et al. reported that the combination of nobiletin with DTX demonstrated strong efficacy and inhibitory effects in comparison to DTX or CAR alone. These findings propose that nobiletin may be considered as a potent enhancer for the principal chemotherapy agent. In vivo studies have revealed that Nobiletin in combination with DTX significantly reduced the volume of tumor, decreasing by 73.5% in comparison to the control group and 36.2% in comparison to DTX alone. These results indicated that combination therapy with DTX and nobiletin could be an innovative treatment for TNBC patients [75].

In a study conducted by Aghamiri et al., the effectiveness of resveratrol (Res) on the radiosensitivity of 5‐FU in a spheroid culture of the MCF‐7 breast cancer cell line was investigated. This research has demonstrated that the combination of 5‐FU with Res and radiation considerably decreased the colony formation capability of spheroid cells. This observation suggests that Res enhances the radiosensitization of spheroid cells associated with breast cancer through inhibiting hypoxia‐inducible factor‐1‐alpha [76].

In line with the chemopreventive and anticancer properties of baicalin on improving the antitumor impact of 5‐FU on breast cancer, the Ehrlich solid tumor‐mice model revealed that pre‐treatment with baicalin and/or treatment with baicalin and 5‐FU effectively decreased angiogenesis and inflammation. These effects exerted by the suppression of VEGF amplification and NF‐kβ/IL‐1β and an increase in apoptosis through the up‐regulation of apoptotic Bax, pro‐apoptotic p53, and caspase‐3, and the downregulation of anti‐apoptotic Bcl‐2. Therefore, baicalin can be effective as a preventative or supplementary treatment in breast cancer therapy with 5‐FU, especially through inducing apoptotic cell death and decreasing angiogenesis and inflammation [77].

### 1.9 Different Strategies for the Development of Plant‐Derived Anticancer Substances

**FIGURE 5:**
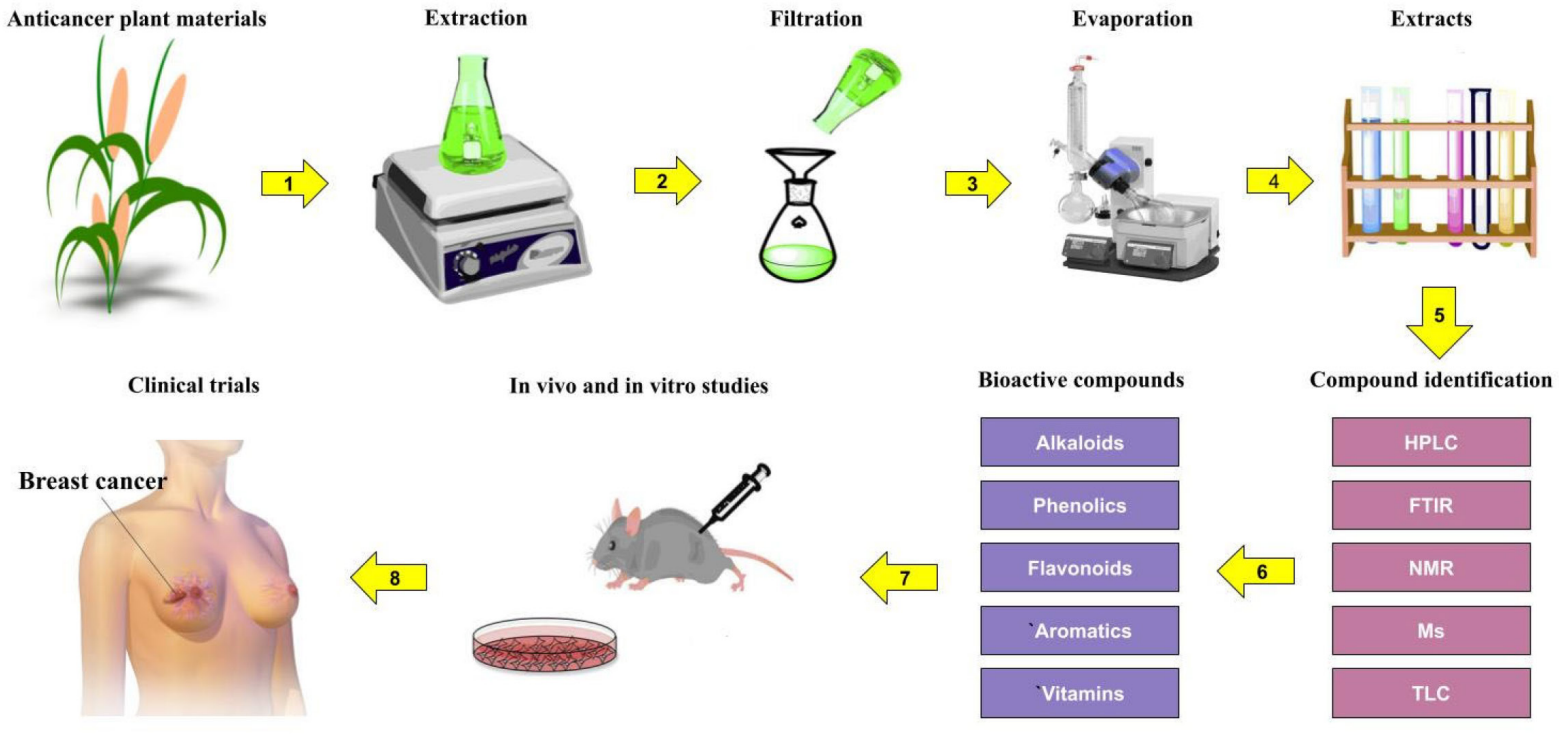
Extraction, characterization, testing, and prospective use of a bioactive chemical as a cancer treatment agent.

The potency of medicinal plants as therapeutic agents depends on the type and quantity of their active compounds, which vary from one species to another, depending on latitude, longitude, altitude, age, climate, and season. Different parts of the plant may have various pharmaceutical effects, which propose them as bioactive compounds in anticancer treatments. Several techniques are used to purify active compounds including combinatorial chemistry, isolation tests, and bioassay‐guided fractionation. The purification of these compounds is carried out in several steps. First, natural extracts (from dry or wet plant materials) with known biological activities are evaluated. Appropriate matrices, such as superdex, sephadex, and silica, are then employed for the fractionation of natural extracts. The fractionated extracts are tested for bioactivity, and then the active fractions are separated using a variety of analytical techniques such as thin layer chromatography (TLC), high performance liquid chromatography (HPLC), Fourier‐transform infrared spectroscopy (FTIR), mass spectroscopy (MS), and nuclear magnetic resonance (NMR). Although these steps are flexible, it is important that the bioactive chemicals have the highest purity, quality, and quantity. This can be achieved by utilizing high‐quality solvents and matrices as well as careful handling. The extracted compounds must be purified before evaluating in vitro or in vivo anticancer effects. Furthermore, it is necessary that more studies are performed on some characteristics of the extracted bioactive compounds such as pharmacokinetics, pharmacodynamics, immunogenicity, metabolic fate, biosafety and side effects, drug interactions, and dose concentration. Figure 5 depicts a thorough planning for the synthesis, characterization, testing, and prospective use of a bioactive chemical as a cancer treatment agent.

### 1.10 Opportunities and Challenges in Plant‐Based Anticancer Therapy

Plants provide an abundant supply of innovative compounds and present a promising new avenue for investigating cancer. Natural products and their derivations are undergoing a revolution because of their reduced toxicity, expediency, cost‐effectiveness, safety, and simplicity in comparison to conventional treatment methods. Natural products are considered promising contenders for the development of anticancer drugs because of the diverse pleiotropic effects on target events. Numerous studies have pointed out that the effects of plant‐derived products are limited to cancer cells while they do not have significant adverse events on healthy cells [78]. Identification of compounds with strong anticancer properties and development of plant‐based medications for cancer treatment might be crucial steps in breast cancer therapy.

Although bioactive compounds have potent anticancer properties, they also have drawbacks that need to be resolved before their application in clinical trials and improved for the approved drugs. The main concerns in regard to the use of these compounds are their poor aqueous solubility, poor penetration into tumors, absorption by normal cells, limited therapeutic activities, and some adverse events [79, 80]. Today, the uses of colchicine, camptothecin, and derivatives of podophyllotoxin are limited because of their side effects. In addition, some anticancer compounds like vinca alkaloids have a limited impact and are usually employed in conjunction with other medications [81]. Further challenges in the discovery and development of new anticancer agents are associated with their extraction, synthesis, optimization, and characterization. New developments in analytical technology and computational methodologies are anticipated to facilitate the identification of new compounds, improve their extraction, and decide on their chemical synthesis or modifications.

## 2 Conclusion and Future Direction

Breast cancer is one of the most difficult tumors to treat, responsible for a large number of cancer‐related fatalities. Cancer has been treated for many years using surgery, radiation, hormone medication, and chemotherapy. Unfortunately, many treatment alternatives are not effective owing to significant side effects and multidrug tolerance/resistance. It is established that plant‐derived compounds have significant anticancer potential and might be quite advantageous using a supplemental therapeutic technique in cancer treatment. These compounds are classified as alkaloids, terpenoids, flavonoids, polyphenols, and vitamins; which can be used in daily diet and have a high potential to treat breast cancer. Natural products have the ability to reduce breast cancer severity, inhibit malignant cell proliferation, and alter cancer‐related pathways. They are able to inhibit Wnt/β‐catenin, NF‐κβ, Notch, and Hedgehog signaling pathways, which participate in breast cancer cell self‐renewal. This review discusses various key plant‐derived compounds and their mechanisms of action, which may be quite effective against breast cancer. Incorporation of natural plant products into breast cancer therapy offers a hopeful approach, which can improve treatment results. Although this study discusses the wide range of plant‐derived chemicals and their impacts on breast cancer, further studies are required to investigate suitable derivatives offering a number of advantages in terms of biological availability and efficacy. Several issues must be addressed in future studies, including the effectiveness of particular compounds, their mechanisms of action, and standardized formulations to validate and incorporate these treatments into mainstream cancer care. The low bioavailability of natural substances usually limits their efficacy. Therefore, further studies must focus on drug delivery systems that can resolve the compound's pharmacokinetic problems and assess the potential for achieving effective local concentrations of plant‐derived products to define their efficacy. It is of utmost importance to prioritize activities aimed at conserving and sustainably using these plant resources. Preservation of natural ecosystems and avocation for ethical use guarantee the conservation of these invaluable treatments for future generations. It seems that the inherent harmony between traditional knowledge and contemporary treatment is needed to reduce the increasing issues of breast cancer worldwide.

## Author Contributions

**Amin Moradi Hasan‐Abad:** validation (equal), writing – original draft (equal). **Ali Sobhani‐Nasab:** supervision (equal), writing – review and editing (equal). **Amir Atapour:** conceptualization (equal), writing – original draft (equal). **Hossein Motedayyen:** investigation (equal), supervision (equal). **Reza ArefNezhad:** project administration (equal), validation (equal).

## Ethics Statement

The authors have nothing to report.

## Consent

The authors have nothing to report.

## Conflicts of Interest

The authors declare no conflicts of interest.

## Data Availability

All data generated or analyzed during this study are included in this published article.
